# Efficacy, safety and prognostic factors in patients with refractory metastatic colorectal cancer treated with trifluridine/tipiracil plus bevacizumab in a real-world setting

**DOI:** 10.1038/s41598-022-18871-9

**Published:** 2022-08-26

**Authors:** Nieves Martínez-Lago, Teresa Calleja Chucla, Beatriz Alonso De Castro, Rafael Varela Ponte, Cristina Reboredo Rendo, Martin Igor Gomez-Randulfe Rodriguez, Sofia Silva Diaz, Begoña Graña Suarez, Juan de la Cámara Gomez, Fernando Busto Fernández, María Mateos Salvador, Margarita Reboredo Lopez

**Affiliations:** 1 Medical Oncology Department, University Hospital A Coruña. Biomedical Research Institute (INIBIC, A Coruña), Xubias de Arriba, 84, 15006 A Coruña, Galicia Spain; 2 Pharmacy Department, University Hospital A Coruña, A Coruña, Spain; 3 Radiology Department, University Hospital Santiago de Compostela, Santiago de Compostela, A Coruña, Spain

**Keywords:** Cancer, Oncology, Cancer, Colorectal cancer

## Abstract

We evaluated the efficacy and safety of trifluridine/tipiracil (TAS-102) plus bevacizumab in treating refractory metastatic colorectal cancer (mCRC) in a retrospective, observational study. Patients refractory or intolerant to standard therapies received TAS-102 (30–35 mg/m^2^ twice daily on days 1–5 and days 8–12 every 28 days) plus bevacizumab 5 mg/kg on days 1 and 15. Clinical and pathological characteristics, overall response rate (ORR), disease control rate (DCR), overall survival (OS) and progression-free survival (PFS) data were collected and analysed. Thirty-five patients were treated from July 2019 to October 2021 (median age 64 years). The majority of patients (68.6%) were receiving TAS-102 plus bevacizumab as third-line treatment. Patients received a median of 4 (range 2–15) cycles of treatment. Among 31 patients evaluable for response (88.6%), ORR and DCR were 3.2% and 51.6%, respectively. After a median 11.6 months’ follow-up, median PFS was 4.3 (95% confidence interval [CI] 3.4–5.1) months and median OS was 9.3 (95% CI 6.6–12.1) months. The most common grade 3–4 toxicities were neutropenia, asthenia and nausea/vomiting, and there were no treatment-related deaths. This real-world study confirms the efficacy and safety of TAS-102 plus bevacizumab in patients with refractory mCRC.

## Introduction

Colorectal cancer (CRC) is the second highest cause of cancer-related death worldwide, with an estimated 1.8 million new cases and > 880,000 deaths in 2018^1^. Evidence-based guidelines recommend cytotoxic chemotherapy (e.g. oxaliplatin, irinotecan or fluoropyrimidines) as first- and second-line treatment in patients with metastatic CRC (mCRC), with the addition of anti-epidermal growth factor receptor (EGFR) agents (i.e. cetuximab or panitumumab) in those with wild-type *RAS* tumours or the anti-vascular endothelial growth factor (VEGF) agent bevacizumab^2^. However, a high proportion of patients develop progressive disease (PD) after receiving standard chemotherapy, with > 40% receiving at least three lines of treatment^3^. Treatment recommendations now include the multi-kinase inhibitor regorafenib and trifluridine/tipiracil (TAS-102) as third-line treatment options in these patients^2^.

The efficacy and safety of TAS-102, a combination of a thymidine-based nucleic acid analogue (trifluridine) and a thymidine phosphorylase inhibitor (tipiracil), have been demonstrated in clinical studies of patients with previously treated mCRC, with significantly improved overall survival (OS) compared with placebo^4–6^. An exploratory analysis of the RECOURSE clinical study^4^ identified patients with low tumour burden and less aggressive disease (i.e. ≥ 18 months since metastatic disease diagnosis) as having improved survival outcomes with TAS-102 monotherapy^7^.

The survival benefits with TAS-102 monotherapy are modest and there is a need for improved treatment options in patients with refractory mCRC. Several phase I/II and phase II studies have investigated the efficacy and safety of TAS-102 in combination with bevacizumab^8–13^. These studies included the single-arm Japanese C-TASK FORCE study in 25 patients with refractory mCRC, which reported a centrally-assessed median progression-free survival (PFS) of 3.7 months and a median OS of 11.4 months with TAS-102 plus bevacizumab^9^. In a Danish randomised study in 93 patients with refractory mCRC, TAS-102 plus bevacizumab was associated with significantly improved median PFS (4.6 vs 2.6 months, hazard ratio [HR] 0.45, p = 0.001) and median OS (9.4 vs 6.7 months, HR 0.55, p = 0.028) compared with TAS-102 monotherapy^10^.

Previous retrospective studies of Japanese patients with refractory mCRC have also indicated that the TAS-102 plus bevacizumab combination provides significant survival benefits compared with TAS-102 monotherapy in routine clinical practice^14–16^; however, real-world data on the use of TAS-102 plus bevacizumab in non-Asian populations are limited. The aim of this real-world study was to evaluate the efficacy, safety and prognostic factors of TAS-102 plus bevacizumab in patients with refractory mCRC in routine clinical practice in Spain.

## Results

### Population characteristics

Thirty-five patients were treated with TAS-102 plus bevacizumab between July 2019 and October 2021 and were included in this study. Patient characteristics are summarised in Table 1. Patients had a median (range) age of 65 (41–82) years and 31.4% were aged ≥ 70 years. The majority of patients (88.6%) had undergone primary tumour resection, 77.1% had an Eastern Cooperative Oncology performance status (ECOG PS) of 0–1, 80.0% had liver metastases, and 71.4% were diagnosed with metastatic disease ≥ 18 months before starting TAS-102 plus bevacizumab. Previous treatment included anti-VEGF therapy in 94.3% of patients (bevacizumab in 57.1% patients, aflibercept in 8.6%, or both bevacizumab and aflibercept in 28.6% patients); 68.6% were receiving TAS-102 plus bevacizumab as third-line treatment. None of the patients had previously received regorafenib.

**Table 1:** Study population characteristics.

Characteristics	N = 35
**Age, years**	
Median (range)	65 (41–82)
≥70 years, n (%)	11 (31.4)
**Gender, n (%)**	
Male	22 (62.9)
Female	13 (37.1)
**ECOG PS, n (%)**	
0–1	27 (77.1)
2	8 (22.9)
**Tumour location, n (%)**	
Right-sided	4 (11.4)
Left-sided	17 (48.6)
Rectum	14 (40.0)
**Histological grade, n (%)**	
Low grade (G1–G2)	22 (62.9)
High grade (G3)	2 (5.7)
Unknown	11 (31.4)
***RAS/BRAF* mutational status, n (%)**	
*RAS/BRAF* wild type	16 (45.7)
*RAS* mutated	17 (48.6)
*BRAF* mutated	2 (5.7)
**Mismatch repair protein expression, n (%)**	
Conserved	35 (100)
**Tumour presentation, n (%)**	
Synchronous	23 65.7)
Metachronous	12 (34.3)
Primary tumour surgery, n (%)	31 (88.6)
Previous anti-VEGF therapy, n (%)	33 (94.3)
Bevacizumab, %	57.1
Aflibercept, %	8.6
Both, %	28.6
**Line of TAS-102 + bevacizumab treatment, n (%)**	
3	24 (68.6)
4	4 (11.4)
≥ 5	7 (20.0)
Liver metastases, n (%)	28 (80.0)
**No. of metastatic locations, n (%)**	
< 3	22 (62.9)
≥ 3	13 (37.2)
**Time from metastatic disease diagnosis, n (%)**	
< 18 months	10 (28.6)
≥ 18 months	25 (71.4)
**Tabernero prognostic classification, n (%)**	
Best	3 (8.6)
Good	12 (34.3)
Poor	20 (57.1)

*ECOG PS* Eastern Cooperative Oncology Group performance status, *G* grade, *No.* number, *TAS-102* trifluridine/tipiracil, *VEGF* vascular endothelial growth factor.

TAS-102 was started at a reduced dose (30 mg/m^2^) in seven patients (20.0%) and no patients started bevacizumab at reduced doses. Prophylactic granulocyte colony-stimulating factor (G-CSF) treatment was administered to five patients (14.3%). Patients received a median of 4 cycles of TAS-102 plus bevacizumab (range 2–15 cycles).

### Efficacy

In total, 31 of 35 patients (88.6%) were evaluable for response; two patients (5.7%) were not evaluable due to an early death, and two patients (5.7%) had response assessment pending at the time of the analysis. After a median follow-up of 11.6 months, 15 patients (48.4%) had PD, one (3.2%) had achieved partial response (PR) and no patients had complete response (CR) (Table 2). The overall response rate (ORR) and disease control rate (DCR) were 3.2% and 51.6%, respectively.

**Table 2:** Response rate.

Response, n (%)	N = 31
**Best overall response**	
CR	0
PR	1 (3.2)
SD	15 (48.4)
PD	15 (48.4)
ORR (CR + PR)	1 (3.2)
DCR (CR + PR + SD)	16 (51.6)

*CR* complete response, *DCR* disease control rate, *ORR* overall response rate, *PD* progressive disease, *PR* partial response, *SD* stable disease.

**Figure 1:**
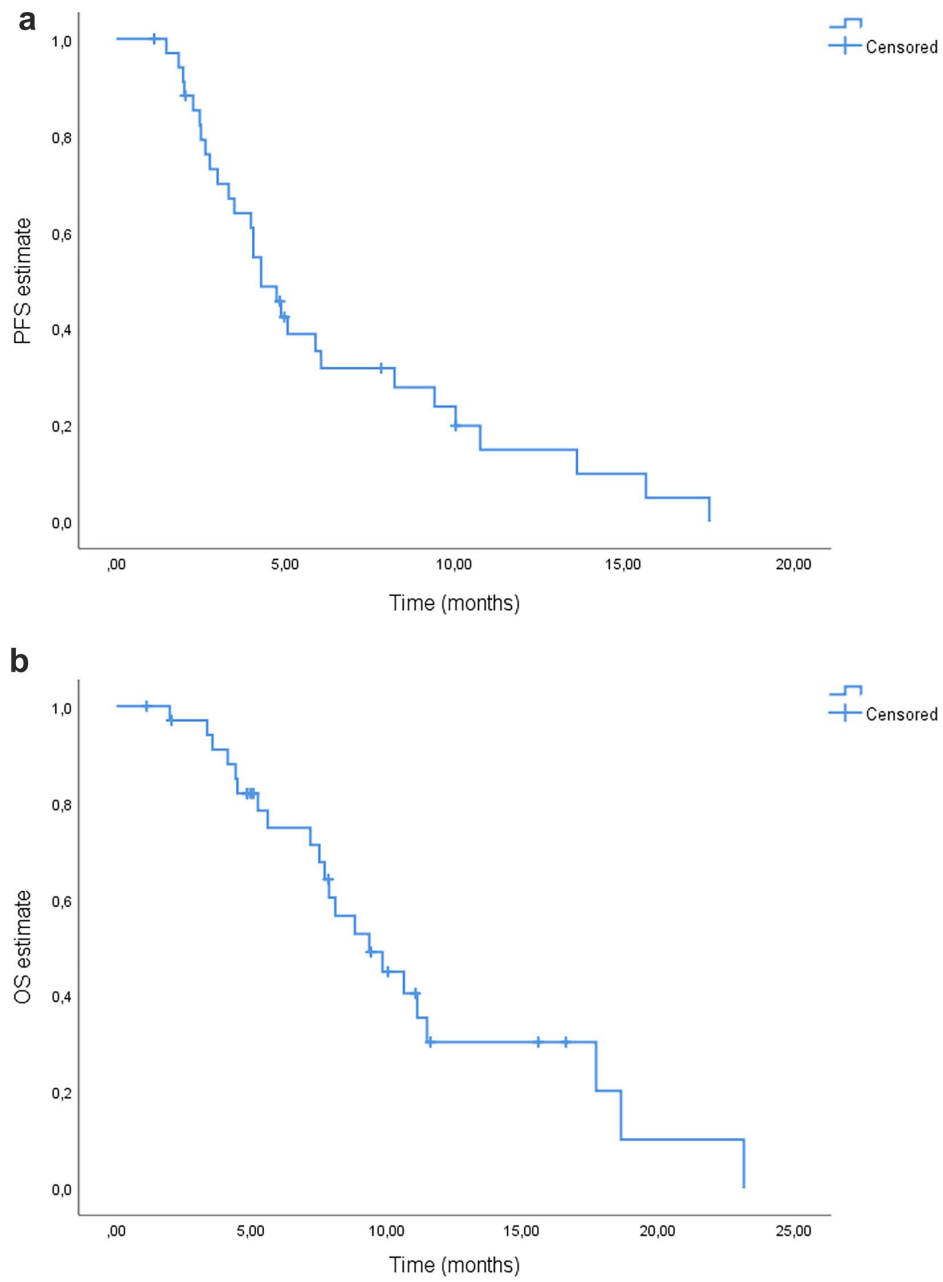
Kaplan–Meier curves for (**a**) progression‐free survival (PFS) and (**b**) overall survival (OS).

Based on Kaplan–Meier estimates, patients had a median PFS of 4.3 months [95% confidence interval (CI) 3.4–5.1 months] (Fig. 1a) and a median OS of 9.3 months (95% CI 6.6–12.1 months) (Fig. 1b).

In the univariate regression analysis, prognostic factors associated with significantly improved OS were the absence (vs presence) of peritoneal metastases and grade 1–2 (vs grade 3) tumour histological grade (Table 3). The absence of peritoneal metastases was also associated with significantly improved PFS, whereas < 3 (vs ≥ 3) metastatic sites was associated with significantly worse PFS.

**Table 3:** Univariate analysis of prognostic factors for progression-free and overall survival.

Characteristic	PFS, months	HR (95% CI)^a^	p-value^a^	OS, months	HR (95% CI)^a^	p-value^a^
**Gender**						
Male	4.2	1.233 (0.6–2.7)	0.599	8.1	1.186 (0.5–2.9)	0.704
Female	4.7	–	–	11.1	–	–
**ECOG PS**						
0–1	4.7	0.652 (0.3–1.6)	0.336	9.3	0.517 (0.2–1.4)	0.182
2	3.5	–	–	8.1	–	–
**Histological grade**						
G1–G2	4.7	0.424 (0.1–1.9)	0.247	10.6	**0.087 (0.1–0.5)**	**0.001**
G3	2.5	–	–	3.4	–	–
**Tumour presentation**						
Synchronous	4.0	2.391 (0.9–5.7)	0.053	9.3	1.017 (0.4–2.6)	0.971
Metachronous	6.0	–	–	10.6	–	–
**Tumour surgery**						
No	2.8	1.044 (0.4–3.0)	0.937	5.3	1.396 (0.5–4.3)	0.562
Yes	4.3	–	–	9.3	–	–
**Metastatic sites**						
< 3	3.0	**2.286 (1.0–5.1)**	**0.001**	5.2	2.790 (0.9–7.8)	0.06
≥3	8.2	–	–	17.7	–	–
**Liver metastases**						
No	17.5	0.309 (0.1–1.1)	0.069	8.1	0.782 (0.2–2.7)	0.696
Yes	4.0	–	–	9.3	–	–
**Peritoneal metastases**						
No	4.7	**0.393 (0.1–0.9)**	**0.046**	9.3	**0.437 (0.2–1.0)**	**0.07**
Yes	2.8	–	–	5.6	–	–
**Time from metastatic disease diagnosis**						
≥18 months	4.3	0.763 (0.3–1.8)	0.523	9.3	0.871 (0.3–2.3)	0.780
< 18 months	4.8	–	–	17.7	–	–
**Tabernero prognostic classification**						
Best	NR	–	0.069	NR	–	0.071
Good	5.0	–	–	11.1	–	–
Poor	3.5	–	–	7.9	–	–

*CI* confidence interval, *ECOG PS* Eastern Cooperative Oncology Group performance status, *G* grade, *HR* hazard ratio, *NR* not reached, *OS* overall survival, *PFS* progression-free survival.

^a^Significant values are indicated in bold text.

### Safety

The most common adverse events (AEs) of any grade were neutropenia (74.3%), asthenia (65.7%), anaemia (54.8%) and thrombocytopenia (34.3%) (Table 4). The most frequent grade 3–4 AEs were neutropenia (45.7%), asthenia (17.1%) and nausea/vomiting (8.6%). There were no reports of febrile neutropenia and no treatment-related deaths. Neutropenia was managed by reducing the dose of TAS-102 in five patients (38.1%) and administration of G-CSF prophylaxis in five patients (33.3%), while in the other patients, treatment was delayed until recovery. In this study, 37.1% of patients required a dose reduction of TAS-102 (20% required one dose level reduction (–5 mg/m^2^), and 17.1% required two dose level reduction (–10 mg/m^2^) from baseline dose level. No patients required dose reduction or discontinuation of bevacizumab.

**Table 4:** Summary of the most frequent all grade and grade 3/4 adverse events.

AE, n (%)	All grades	Grade 3–4
Neutropenia	26 (74.3)	16 (45.7)
Asthenia	23 (65.7)	6 (17.1)
Anaemia	17 (54.8)	2 (5.7)
Thrombocytopenia	12 (34.3)	2 (5.7)
Diarrhoea	12 (34.3)	2 (5.7)
Hepatic function abnormalities	8 (22.9)	0
Nausea/vomiting	6 (17.1)	3 (8.6)
Bleeding	4 (11.4)	0
Hypertension	2 (5.7)	1 (2.9)
Venous thromboembolism	0	0
Febrile neutropenia	0	0

*AE* adverse event.

## Discussion

In this real-world study of TAS-102 plus bevacizumab treatment in patients with refractory mCRC, efficacy and safety data were generally consistent with those of previous clinical studies, including the Japanese C-TASK FORCE study^9^ and the Danish phase II study^10^. The ORR in our study (3.2%) was slightly higher than that reported in C-TASK FORCE (0% by central assessment)^9^ and the Danish study (2%)^10^, whereas the DCR was slightly lower in our study (51.6%) than in earlier studies (64% and 67%, respectively)^9,10^. In our study, the median PFS (4.3 months) was similar to that reported in the earlier studies (3.7 and 4.6 months, respectively), while the median OS (9.3 months) was similar to that of the Danish study (9.4 months)^10^, but slightly lower than in C-TASK FORCE (11.4 months)^9^.

The efficacy of TAS-102 plus bevacizumab in our study was also generally comparable with that reported in previous real-world retrospective studies of Japanese patients with refractory mCRC, in which the median PFS with TAS-102 plus bevacizumab was 3.7 months^16^ or 4.4 months^14^, and the median OS ranged from 8.6 to 14.4 months^14–16^.

Patients with refractory mCRC often have poor prognosis^17^. In our real-world study, 22.9% of patients had an ECOG PS of 2 and therefore may be more representative of patients with refractory mCRC in routine clinical practice than the previous C-TASK FORCE and Danish clinical studies, which excluded patients with ECOG PS of 2^9,10^. In the previous Japanese real-world studies of TAS-102 plus bevacizumab, the proportion of patients with ECOG PS of 2 (or modified Glasgow prognostic score of 2) was also much lower (1.4–4.8%)^14–16^ than in our study. Therefore, our study indicates that TAS-102 plus bevacizumab continues to be effective in patients with refractory mCRC and poor performance status scores.

In the univariate analysis of prognostic factors for survival, our study showed that OS and PFS were significantly improved in patients without peritoneal metastases, and those with low tumour histological grade had significantly improved OS. However, patients with low tumour burden (< 3 metastatic sites) had significantly worse PFS compared with those with ≥ 3 metastatic sites. Although this result seems counterintuitive, the low tumour burden may be an indicator of treatment intensity or the finding may be a statistical artefact associated with the small population size of our study. Another study found no difference in survival outcomes between patients CRC with three versus four metastatic sites^18^. Moreover, a large database analysis of the correlates of survival showed that the organ affected by metastasis was an important determinant of survival^19^. Further research is needed to determine whether it is the number of metastatic sites or the organs affected by metastases that has the greatest impact on survival outcomes.

Previous studies have identified other baseline prognostic factors associated with improved clinical outcomes with TAS-102 (either as monotherapy or combined with bevacizumab), including modified Glasgow prognostic score^20^, the Tabernero prognostic factors [i.e. low tumour burden, less aggressive disease (≥ 18 months since diagnosis of metastatic disease) and liver metastases]^7^, high lymphocyte-to-monocyte ratio (≥ 3.18)^21^, and the TAS-RECOSMO predictive model [i.e. general status, neutrophil-to-lymphocyte ratio, *KRAS*, *NRAS* and *BRAF* mutation status, carcinoembryonic antigen (CEA) and alkaline phosphatase (ALP) levels, and time since metastatic disease diagnosis]^17^. However, our study did not identify liver metastases or the time since diagnosis of metastasis < 18 months (i.e. the Tabernero factors) as being prognostic of OS or PFS, and we did not examine mutational status, CEA or ALP levels, or lymphocyte-to-monocyte or neutrophil-to-lymphocyte ratios as potential prognostic factors.

In our study, TAS-102 plus bevacizumab was associated with manageable toxicities, with the most common grade 3–4 AEs being neutropenia, asthenia and nausea/vomiting. The incidence of grade 3–4 neutropenia (45.7%) was lower than that reported in the C-TASK FORCE study (72%)^9^ and the Danish study (67%)^10^, and was slightly lower than in previous Japanese real-world studies (48.2–52.4%)^14–16^. Furthermore, no patients developed febrile neutropenia in our study, while the incidence of this event was 16% and 6%, respectively, in C-TASK FORCE and the Danish study^9,10^, and 0–3.3% in the Japanese real-world studies^14–16^. The lower levels of haematological toxicity observed in our study may have been due to the relatively high proportion of patients who received prophylactic G-CSF therapy (14.3%). Of note, several studies have reported that chemotherapy-induced neutropenia with TAS-102 (with or without bevacizumab) is associated with improved survival outcomes^22–24^, which highlights the importance of G-CSF prophylaxis to prevent or manage neutropenia and allow for continued TAS-102 plus bevacizumab treatment without the need for dose reduction.

The limitations of our study include its retrospective, single-arm, single-centre design and its small population size (N = 35). An ongoing international phase III study (SUNLIGHT; NCT04737187) is currently investigating the efficacy and safety of TAS-102 plus bevacizumab versus TAS-102 monotherapy as third-line treatment in patients with refractory mCRC, and has a target enrolment of 490 patients^25^. This open-label, multicentre study aims to further confirm the clinical benefits of TAS-102 plus bevacizumab over TAS-102 monotherapy in a large population of patients with refractory mCRC; results are expected in 2023.

In conclusion, this real-world study confirms the efficacy and safety of TAS-102 plus bevacizumab in patients with refractory mCRC in routine clinical practice, with survival and tolerability outcomes that were generally consistent with previous clinical and real-world studies of patients in this setting.

## Methods

### Study design

This observational, retrospective, single-centre study was conducted at the A Coruña University Hospital in Spain in patients aged > 18 years with a confirmed diagnosis of mCRC who were refractory or intolerant to standard therapies. Previous treatment included fluoropyrimidine, oxaliplatin and irinotecan-based chemotherapy and anti-EGFR agents (in patients with wild-type RAS/BRAF tumours). Eligible patients had received treatment with TAS-102 plus bevacizumab in routine clinical practice between July 2019 and October 2021, including patients who had previously received treatment with antiangiogenic agents (i.e. bevacizumab and/or aflibercept). Patients who had previously received TAS-102 monotherapy or TAS-102 in combination with antiangiogenic agents other than bevacizumab were excluded.

The standard doses administered at A Coruña University Hospital were TAS-102 30–35 mg/m^2^ on days 1–5 and days 8–12 every 28 days plus bevacizumab 5 mg/kg every 14 days. Starting treatment with reduced doses of TAS-102 or administration of prophylactic G-CSF was at the discretion of the treating physician.

The study was approved by the local ethics committee and conducted in accordance with the Declaration of Helsinki. All patients gave their informed consent prior to study inclusion.

### Data collection

Clinical pathological characteristics and treatment data were collected from eligible patients’ medical records, including sex, age and ECOG PS. Disease characteristics included RAS and BRAF mutational status, mismatch repair protein expression, primary tumour location, histological grade, tumour presentation (synchronous or metachronous), the number of metastatic locations (< 3 or ≥ 3), the interval from metastatic disease diagnosis to TAS-102 plus bevacizumab initiation (≥ 18 or < 18 months), and the Tabernero prognostic classification (best, good or poor)^7^, as well as treatment history, including primary tumour resection, metastatic disease resection and previous treatments received. The start date and initial doses of TAS-102 plus bevacizumab, the use of prophylactic G-CSF, the number of cycles received, the response obtained (assessed by Response Evaluation Criteria in Solid Tumors version 1.1 criteria), disease progression and/or survival, toxicities according to Common Terminology Criteria for Adverse Events version 4.0, and any dose delays and/or dose reductions were also collected retrospectively from patient records for analysis.

### Study assessments

OS was defined as the time between treatment initiation and death from any cause. PFS was defined as the interval between treatment initiation and radiological confirmation of disease progression or death from any cause. The ORR was defined as the proportion of patients who achieved CR or PR; the DCR was defined as the proportion of patients who achieved CR, PR, or stable disease for ≥ 6 weeks after treatment initiation.

### Statistical analyses

Statistical analyses were performed using SPSS statistics software version 25.0. The Chi-squared or Fisher’s exact test (depending on the sample size) was used to compare patient clinical and demographic variables. The Kaplan–Meier model was used to estimate median PFS and OS and their 95% CIs. An analysis of potential predictors of PFS or OS was also conducted by comparing the differences between survival curves using univariate logistic regression and the log-rank test with a two-sided significance of < 0.05.

### Institutional review board statement

Approved by Clinical Research Ethic Committee (CEIC) of Galicia, Spain.

## Data Availability

All data are available upon request from the corresponding author.
